# Bovine Animal Model for Studying the Maternal Microbiome, *in utero* Microbial Colonization and Their Role in Offspring Development and Fetal Programming

**DOI:** 10.3389/fmicb.2022.854453

**Published:** 2022-02-23

**Authors:** Samat Amat, Carl R. Dahlen, Kendall C. Swanson, Alison K. Ward, Lawrence P. Reynolds, Joel S. Caton

**Affiliations:** ^1^Department of Microbiological Sciences, North Dakota State University, Fargo, ND, United States; ^2^Department of Animal Sciences, and Center for Nutrition and Pregnancy, North Dakota State University, Fargo, ND, United States

**Keywords:** maternal microbiome, *in utero* microbial colonization, bovine model, developmental origins of health and disease, fetal programming, biomedical research

## Abstract

Recent developments call for further research on the timing and mechanisms involved in the initial colonization of the fetal/infant gut by the maternal microbiome and its role in Developmental Origins of Health and Disease (DOHaD). Although progress has been made using primarily preterm infants, ethical and legal constraints hinder research progress in embryo/fetal-related research and understanding the developmental and mechanistic roles of the maternal microbiome in fetal microbial imprinting and its long-term role in early-life microbiome development. Rodent models have proven very good for studying the role of the maternal microbiome in fetal programming. However, some inherent limitations in these animal models make it challenging to study perinatal microbial colonization from a biomedical standpoint. In this review, we discuss the potential use of bovine animals as a biomedical model to study the maternal microbiome, *in utero* microbial colonization of the fetal gut, and their impact on offspring development and DOHaD.

## Introduction

The completion of the NIH Human Microbiome Project and advances in functional and translational microbiome research using meta-omics approaches have further enhanced our fundamental understanding of the role of the human microbiome in defining health and disease (Turnbaugh et al., 2007; Shreiner et al., 2015; Integrative HMP (iHMP) Research Network Consortium, 2019). The microbial communities residing within the gastrointestinal tract are vital to human health not only for their ability to digest and utilize a variety of nutrients in the diet, and produce bioactive compounds such as volatile fatty acids, vitamin K, and others, but also because of their ability to influence both infectious and non-infectious metabolic diseases including obesity (Boulangé et al., 2016), diabetes (Li et al., 2020), cardiovascular diseases (Tang et al., 2017), cancer, autoimmune (Zhang et al., 2020), and neurological disorders (Wang et al., 2019).

The neonatal gut harbors a low-diversity of microbiota at birth; however, from birth, the gut microbial community undergoes developmental, transitional, and stable phases of progression before converging toward an adult-like microbiota by the end of the first 3–5 years of life (Rodríguez et al., 2015; Stewart et al., 2018). Increasing evidence suggests that the early-life microbiome is involved in the regulation of immune, endocrine, and metabolic developmental pathways (Robertson et al., 2019), and thus, normal development of the early-life microbiome is critical to health and well-being later in life (Arrieta et al., 2014; Tamburini et al., 2016).

Emerging evidence derived from both human and vertebrate animal models suggests that the maternal microbiome during pregnancy is not only important to maintain the health of the pregnant mother and meet the increased metabolic demands of her developing fetus, but it also has an extended impact on the offspring’s development and health. The potential involvement of the maternal microbiome in the Developmental Origins of Health and Disease (DOHaD) has recently begun to be better appreciated (Stiemsma and Michels, 2018; Calatayud et al., 2019; Codagnone et al., 2019b). In addition, emerging evidence derived from the detection of microorganisms in meconium (Nagpal et al., 2016; He et al., 2020), fetal fluids (Urushiyama et al., 2017; Stinson et al., 2019), and fetal intestines (Rackaityte et al., 2020; Mishra et al., 2021) suggests that microbial seeding of the infant intestine may begin *in utero*. Given that maternal microbiota can be the primary inoculant sources for the pioneer fetal microbiota, and that the maternal gut microbiota during pregnancy can modulate fetal metabolism and neurodevelopment (Kimura et al., 2020; Vuong et al., 2020), understanding the role of the maternal microbiota, and feto-maternal microbial crosstalk in fetal programming and offspring microbiome development is critical for developing strategies to enhance maternal gut microbiome-mediated health in pregnant women, and improve fetal development and offspring health.

Due to increasing research, interest has recently been placed in uncovering the potential role of the microbiome in DOHaD, and the laboratory mouse model has been used to elucidate the involvement of the maternal gut microbiota in programming of fetal metabolic (Kimura et al., 2020) and nervous system development (Vuong et al., 2020). However, the ethical and legal restrictions associated with human subjects, and the inherent limitations of the rodent animal models (e.g., difference in reproductive physiology, microbial ecology, gestational age, and pregnancy) make it compelling to use more relevant large animal models.

Cattle have a large proportion of singleton pregnancy and as a similar gestation period (280 days, 40 weeks) as humans and are colonized by microbiotas that are biogeographically and phylogenetically more identical to the human microbiota compared to rodents. Investigation of maternal microbiota and perinatal microbial colonization using a bovine animal model may provide more relevant information than other animals for maternal, fetal, and pediatric medicine. In this review, we will provide a brief overview of the emerging evidence highlighting the potential and extended role of the maternal microbiome in fetal programming and offspring development. Then, we will discuss potential use of the bovine animal model as a biomedical research model to explore the maternal gut microbiome, *in utero* microbial colonization, and their impact on fetal programming and early-life microbiome development. We will outline hypothetical approaches that can be applied with bovine animal models to provide mechanistic understanding of the link between the maternal microbiota and fetal programming and offspring development. Then, we will close the review by acknowledging the potential challenges associated with using bovine models to study the maternal microbiome, *in utero* microbial colonization, and their implications in DOHaD.

## Maternal Microbiome During Gestation and Its Potential and Extended Role in Offspring Development

### Maternal Microbiota Changes During Pregnancy

According to the data derived from rodent and human models, the maternal gut microbiota undergoes profound changes over the course of pregnancy (Collado et al., 2008; Koren et al., 2012; Nuriel-Ohayon et al., 2016; Smid et al., 2018). As pregnancy progresses from the 1st to the 3rd trimester, the maternal gut microbiota becomes less diverse as characterized by an increased abundance of *Proteobacteria* and *Actinobacteria* (Koren et al., 2012), and greater microbial density (Collado et al., 2008). When transferred into germ-free mice, the *Proteobacteria*-dominated maternal microbiota from the 3rd trimester resulted in increased fat deposition, inflammation, and insulin insensitivity compared to the 1st trimester microbiota (Koren et al., 2012). This observation indicates that changes in the gut microbiota during pregnancy may be an adaptive process that has evolved over time to meet the increased metabolic demands of the developing fetus (Smid et al., 2018; Codagnone et al., 2019a).

Maintenance of a successful pregnancy relies on molecular (Lash, 2015) and immunological (Arck and Hecher, 2013; Ander et al., 2019) crosstalk at the feto-maternal interface. However, the mechanisms underlying the regulation of the maternal gut microbiota response to pregnancy are yet to be determined. It is reasonable to speculate that interplay between different cells and molecules at the feto-maternal interface may be involved in the regulation of the maternal gut microbiome response during pregnancy. Additionally, any miscommunication between the fetus and mother may lead to dysregulation of maternal gut microbial assembly, which may result in compromised embryonic/fetal development. Thus, in addition to dietary and lifestyle factors (e.g., antibiotics) that can have a direct impact on the maternal gut microbiota during pregnancy, it is important to investigate how feto-maternal crosstalk influences the maternal and fetal gut microbiomes because dysregulation of the pregnancy-associated gut microbiota development may have detrimental effects on maternal metabolism during pregnancy and both *in utero* and postnatal offspring development.

### Evidence Suggesting the Involvement of the Maternal Microbiota in Fetal Programming

Whereas the role of maternal nutrition in programming of offspring metabolic, immune, and nervous system development has been relatively well documented in humans and food-producing animals including cattle (Palmer, 2011; Caton et al., 2019), the potential involvement of the maternal microbiome in the DOHaD has recently begun to be better appreciated (Stiemsma and Michels, 2018; Calatayud et al., 2019; Codagnone et al., 2019b). It has been believed that maladaptive alterations of the maternal microbiota could indirectly influence fetal development, and these effects may get transmitted to progeny, subsequently resulting in the development of altered microbiota in the offspring (Calatayud et al., 2019). Undesired outcomes resulting from maternal microbiota changes on offspring microbiome development include increased offspring susceptibility to the development of metabolic disorders, respiratory infection, and diabetes (Calatayud et al., 2019; Yao et al., 2020).

One of the underlying mechanisms by which the maternal microbiota influences offspring metabolic programming has recently been uncovered in a mouse study (Kimura et al., 2020). Kimura et al. (2020) demonstrated that the maternal gut microbiota modulates metabolic programming of offspring beginning at the embryonic stage. Short-chain fatty acids (SCFAs) derived from the maternal gut microbiota reach the placenta and are transferred to the developing embryos, where the SCFA propionate mediates insulin levels and sympathetic nervous system development through G-protein-coupled receptor signaling pathways. In addition, the role of the maternal gut microbiota in developmental origins of brain health and disease has been documented (Kim et al., 2017; Codagnone et al., 2019b). Another recent mouse study demonstrated that the maternal microbiome modulates fetal neurodevelopment by regulation of maternal serum and fetal brain metabolites during pregnancy (Vuong et al., 2020). Although the influence of maternal gut microbiota during pregnancy on immune, metabolic, and brain development of offspring has been documented, how the maternal gut microbiota influence fetal and offspring microbiome development remains largely undefined. Considering the increasing evidence showing the importance of the maternal microbiota in developmental programming shown in rodent animal models, and increased appreciation of the role of the bovine microbiome in defining cattle health and productivity, exploring the role of the maternal microbiota in fetal programming and offspring development may provide important information to improve cattle health and feed efficiency and has implications for developmental programming in humans.

### Emerging Evidence Suggesting the Existence of *in utero* Microbial Colonization

Although the concept of *in utero* microbial colonization is still controversial, with many still supporting the “sterile-womb hypothesis” that infant microbiome acquisition occurs only during and after birth (Perez-Muñoz et al., 2017; de Goffau et al., 2019), very recent studies (He et al., 2020; Rackaityte et al., 2020) have provided convincing evidence supporting the former hypothesis. Rackaityte et al. (2020) were able to culture viable bacteria (*Micrococcaceae* and *Lactobacillus* strains) from the human fetal intestine. Likewise, He et al. (2020) reported that seeding of the meconium microbiota is partially contributed by the microorganisms found in amniotic fluid. Furthermore, bacterial presence in the intestinal lumen of 14- and 18-week-old human (i.e., early second trimester) fetuses has recently been demonstrated by sequencing, imaging, and culture-based approaches (Mishra et al., 2021).

Emerging evidence derived from bovine animal research also supports the *in utero* microbial colonization hypothesis and challenges the dogma that ruminal colonization by various microbes starts only at or after birth (Abecia et al., 2014; Guzman et al., 2015). Results of a study that used 16S rRNA sequencing, qPCR, and culturing to characterized the bacterial load and composition of amniotic fluid and meconium of near full-term calves delivered *via* caesarian section revealed that *in utero* maternal-fetal bacterial transmission may occur before birth in calves (Husso et al., 2021). In addition, Guzman et al. (2020) investigated the presence of microbiota in five different locations along the length of the GIT and amniotic fluid obtained from calf fetuses at 5, 6, and 7 months of gestational age (the average length of bovine gestation is 280 days) using both molecular- and culture-based approaches. The 16S rRNA gene amplicon sequencing results showed that relatively diverse and distinct bacterial and archaeal communities were present in the fetal GIT and amniotic fluid, and that microbial richness varied different locations within GIT. Quantitative PCR results also indicated that the total bacterial abundance may increase in GIT as the gestational age increases. The authors were also able to culture viable bacterial isolates from intestinal fluid samples. The observations of Guzman et al. (2020) are further supported by our own data, which suggested that microbial colonization of fetal intestine may take place within the first 12 weeks of gestation in cattle (Amat et al., 2021a). These studies together provide sequencing and culture-based evidence to support that the intestine of the calf fetus is not sterile and colonization by pioneer microbes may occur during gestation. When coupled with emerging support for the potential role of the microbiome in the DOHaD, further research to evaluate the timing and mechanisms involved in the initial colonization of the fetal/infant gut is critical to a comprehensive understanding of the development of the early-life gut microbiome (Chu et al., 2017; Walker et al., 2017) and longer-term impacts on offspring growth, health, and well-being.

## Bovine Animal Model to Study the Maternal Microbiome, *in utero* Microbial Colonization, and Their Role in Fetal Programming and Offspring Development

### General Consideration

Livestock have contributed significantly to biomedical advancements from the earliest stages of biological sciences (Reynolds et al., 2009; Polejaeva et al., 2016). Unfortunately, the contribution of livestock to past, current, and potentially future advancements in our understanding of biology and biomedical approaches to improving health and well-being are often overlooked (Ireland et al., 2008; Reynolds et al., 2009). Recently, Hamernik (2019) organized an entire issue of the journal *Animal Frontiers* around the concept that “*Farm animals are Important Biomedical Models*.” Specifically relevant to the current review, bovine are exceptionally robust biomedical models in multiple areas of research (Ireland et al., 2008; Abedal-Majed and Cupp, 2019; Hamernik, 2019; Zhao et al., 2019). Research areas include muscle development and metabolism (Zhao et al., 2019), developmental programming (Caton et al., 2019; Diniz et al., 2021a,b), digestive and metabolic systems (Dänicke et al., 2014), immune system development (Guzman and Montoya, 2018), reproductive physiology (Malhi et al., 2005), toxicology (Santos et al., 2014), *in vitro* fertilization (Ménézo and Hérubel, 2002), epigenetics, nervous system biology (Gurda and Vite, 2019), cardiovascular disease (Tsang et al., 2016), infectious disease (Birch et al., 2018), as well as vaccine development (Gershwin et al., 1994).

Bovine as a model to study the role of the maternal microbiome in developmental programming has to date been overlooked but may, in fact, be an ideal animal model for these types of studies (Table 1). Bovine have a long gestational length (similar to humans) usually carry singleton pregnancies, provide an opportunity for instrumentation and an abundance of samples (for immediate research and long-term storage for potential future investigations), the physiology of pregnancy and infancy are relatively well studied, the use of assisted reproductive technologies including *in vitro* fertilization is well established in livestock and embryos from these technologies make particularly poor pregnancies just as in humans (Reynolds et al., 2014), and they allow for experiments that could not be done in humans because of ethical concerns (Reynolds and Vonnahme, 2017). In addition, dedicated bovine facilities and personnel are often present for production-level research traditionally performed with US Department of Agriculture (USDA) or Agriculture Experimentation Station partners. Advantages associated with bovine models for investigating the interrelationships of the microbiome and developmental programming outcomes contrast sharply with the inherent limitations of rodent animal models (shorter gestational age, immaturity of pups at birth, small body size of both dams and fetuses, and litters vs. singleton offspring) and may make bovine models a superior choice to study *in utero* microbial colonization and its relationship to developmental and metabolic outcomes.

**Table 1:** Summary of reproductive characteristics and life cycle of humans, cattle, sheep, and rodents.^1^

Reproductive characteristic	Women	Cattle (Cow)	Sheep (Ewe)	Pig (Saw)	Rodent animals (Mice)
Duration of gestation (days)	278–282	278–282	142–148	114–116	21
Ovulatory cycle (days)	24–30	17–24	13–19	18–24	4–6
Days of gestation that fetal follicle assembly occurs	133	142	100	70–90	Shortly after birth
Ovulations per cycle	1	1	1–3	15–30	12–14
Length of follicular phase (days)	12–14	2–3	2–3	5–7	1–3
Length of luteal phase (days)	14–16	15–18	12–14	13–15	Depends on whether female engages in copulation
Diameter of ovulatory follicle (mm)	18–20	15–20	5–7	10–12	0.9–1.1
Age of first mating		15–18 months	7–9 months	6–7 months	6–8 weeks
Age for first birth		24–27 months	12–14 months	9–10 months	7–9 weeks
Number of births per year		1	1–2	2	5–10

1 The information presented in this table is adapted from Ding et al. (2010); Talafha and Ababneh (2011); Bazer et al. (2012); Abedal-Majed and Cupp (2019); and Nowak et al. (2020).

### Bovine Reproductive Physiology Is More Representative to the Human Reproductive Physiology Compared to Other Farm and Rodent Animal Models

**Figure 1:**
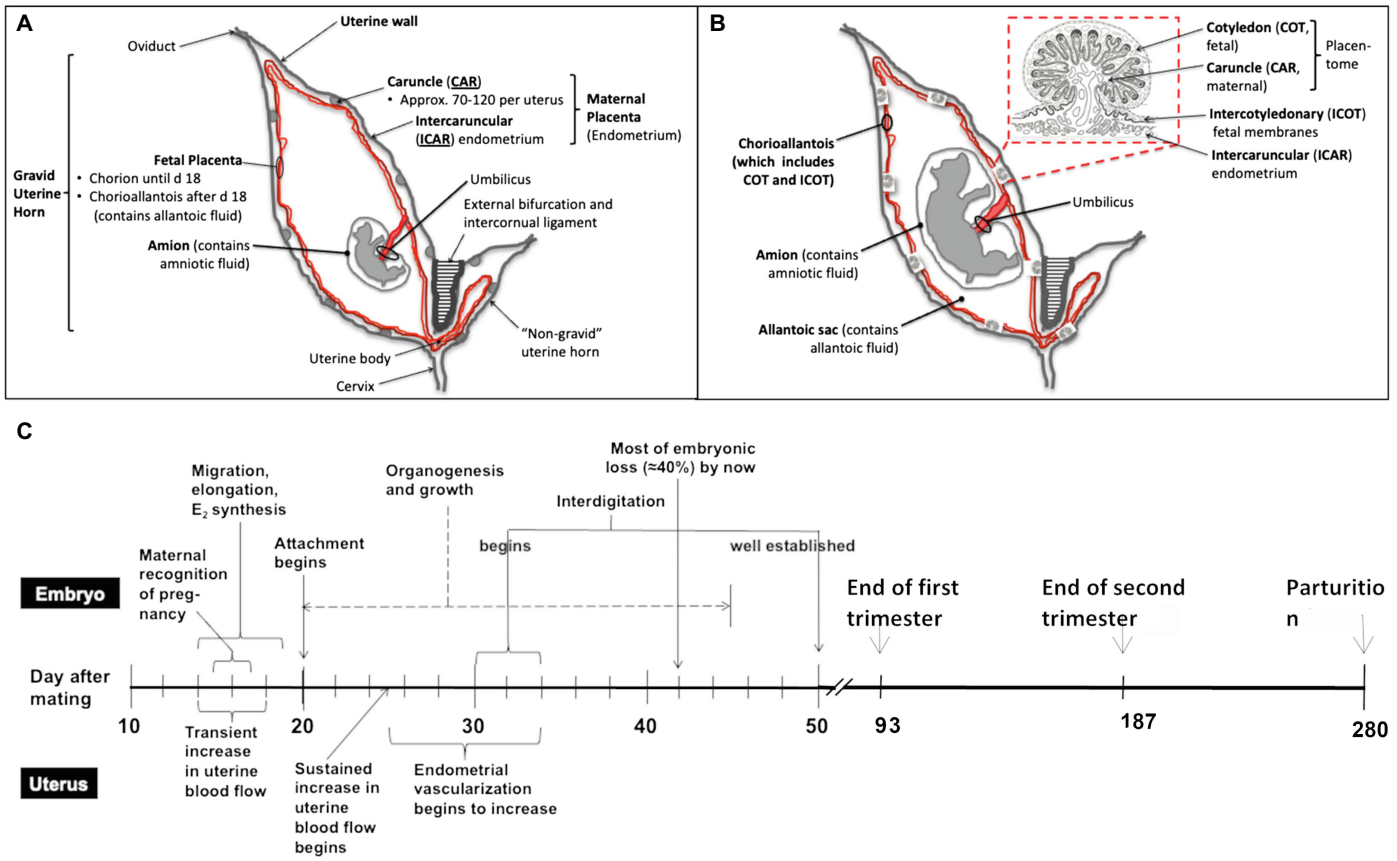
Schematic overview of the bovine reproductive tract, different developmental stages of utero-placenta and fetal development (**A**, day 25–30 after mating; **B**, mid to late pregnancy), and the timeline of placental and embryonic/fetal development during the entire pregnancy in cattle **(C)**. Timeline modified from Caton et al. (2020). As shown in **(A)** and **(B)**, the fetus and placenta (both the maternal and fetal portions) are contained in the gravid uterine horn (normally present in only one uterine horn as cattle typically have singleton pregnancies). Over the course of pregnancy, from early **(A)** to mid to late **(B)** pregnancy, the fetus develops (organogenesis is completed by about day 45–50 of pregnancy), and the fetal organs and placenta grow and mature. As mentioned, the placenta consists of both fetal (cotyledon and intercotyledonary fetal membranes) and maternal (caruncle and intercaruncular endometrium) components.

**Figure 2:**
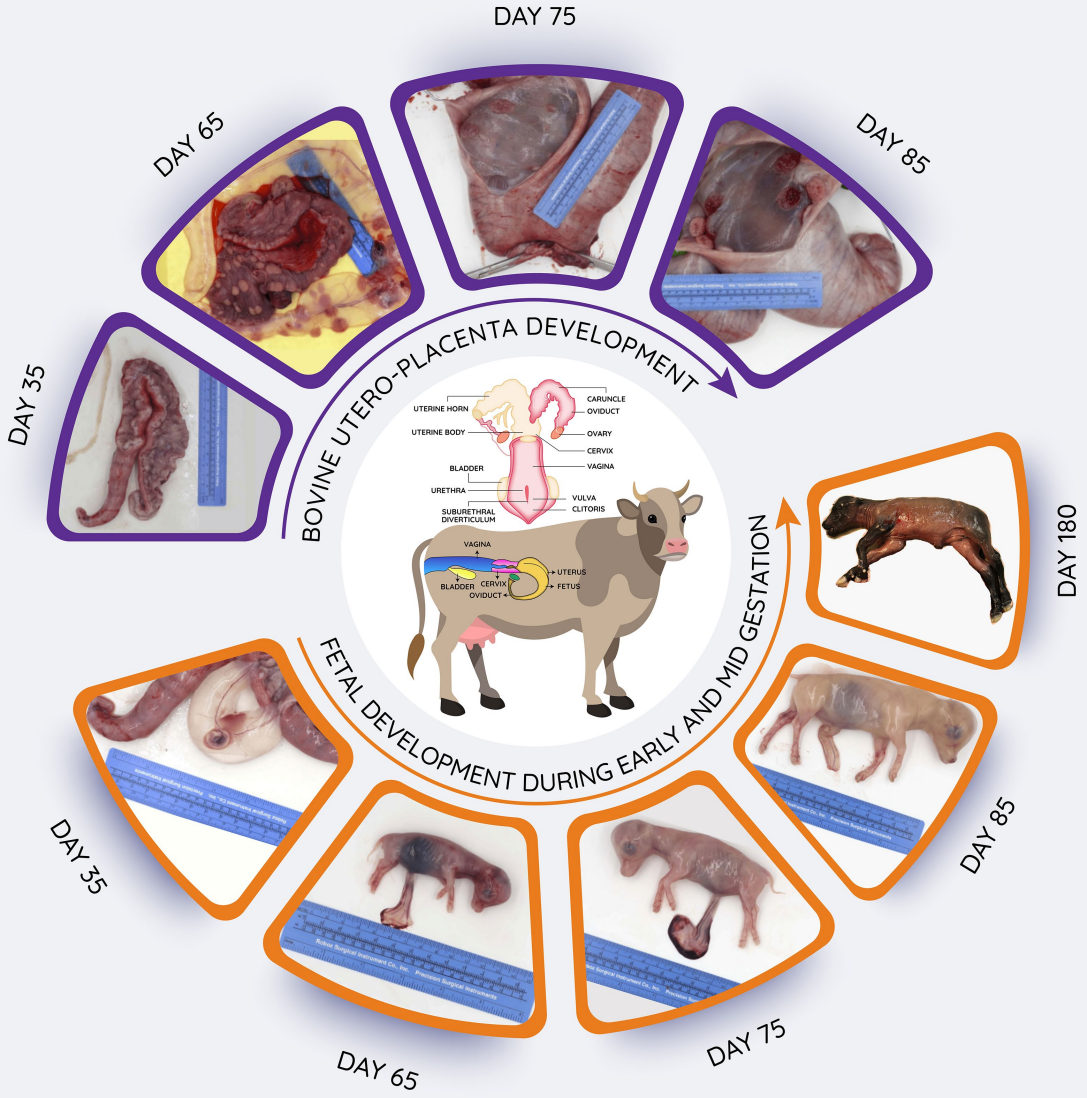
Schematic overview of bovine reproductive tract, and images of utero-placental development from 35 to 85 days after mating as well as fetuses obtained during early (day 35) to late (day180) gestation. Length of gestation is around 280 days. In the upper images, the uterine horns have been opened to reveal the utero-placental surfaces. In the lower images, the uterus has been opened to reveal the fetus in its fetal membranes (chorioallantois and amnion; day 35) or the fetuses have been removed to illustrate their continued growth and development.

Relevant to this review, reproductive characteristics of cattle are similar to those of humans (Table 1). In both cattle and human, reproductive function is regulated by hypothalamic gonadotropin-releasing hormone, which stimulates release of pituitary follicle-stimulating hormone and luteinizing hormone, both of which are trophic for the ovaries (Hunzicker-Dunn and Mayo, 2015; McCardle and Roberson, 2015; McKenna, 2015; Pangas and Rajkovic, 2015; Senger, 2015). Although the bovine reproductive tract anatomy differs from that of humans, the differences are only slight and have to do primarily with shape (Figures 1, 2). More specifically, the reproductive tract of cattle and other ungulates (hoofed animals) has two uterine cornua, or horns, which communicate *via* a common uterine body, and is therefore termed bicornuate, whereas that of the human and other primates lacks defined cornua, and is therefore termed simplex (Ramsey, 1982; Senger, 2015). However, both bovine and human reproductive tracts consist of ovaries, oviducts, uterus, cervix, and vagina (Ramsey, 1982; Senger, 2015).

Similarly, the placentas of cows and humans differ in shape, and humans have a single placental disk, whereas cattle and other artiodactyls (even-toed ungulates, including cattle, sheep, deer, goats, buffalo, bison, antelope, and giraffes) have multiple (in cattle, 60–100 or so) “placentomes” distributed over the surface of the outer fetal membrane, or chorioallantois, consisting of the fetal cotyledons, and maternal caruncles, which interdigitate extensively (Ramsey, 1982). More importantly for this discussion, the placentas of both cattle and humans are similar in function. For an example, they both provide for transport of nutrients, respiratory gases and wastes between the maternal and fetal systems, and at least on the fetal side of the placenta which is consist of numerous, highly vascular villi (Figure 1) known as “vascular, mesodermal allantoic villi” (Mossman, 1987). Moreover, gestation length is similar between domestic cattle and humans, being approximately 38 weeks from fertilization to birth in humans and 40 weeks in cattle (McLaren, 1972).

### Cattle Microbiome and Its Potential Applications to Study the Role of Microbiome in DOHaD

**Figure 3:**
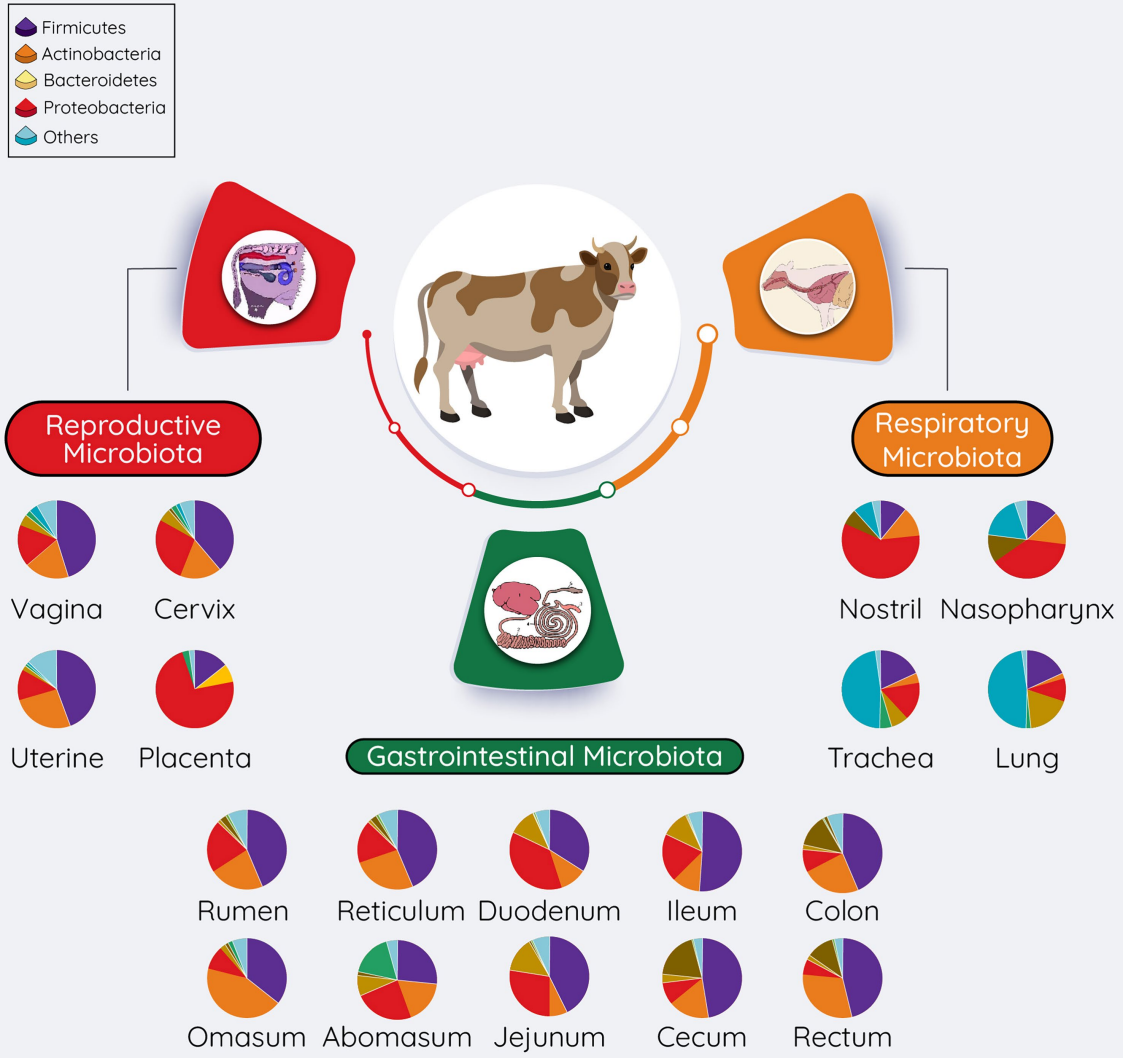
Overall summary of the microbial communities residing within the respiratory, gastrointestinal, and reproductive tracts of cattle. The pie chart represents the most relatively abundant bacterial phyla. The relative abundance of these phyla present in pie charts was adapted from McMullen et al. (2020a) (respiratory tract), Mao et al. (2015), and Holman and Gzyl (2019) (gastrointestinal tract), reproductive tract (Amat et al., 2021b; vagina; Wang et al., 2018; Cervix; Quereda et al., 2020; Uterine).

A diverse microbiota presents in bovine respiratory, gastrointestinal, and reproductive tracts (Figure 3). Overall microbial diversity and composition of many of these body sites share similarity to the microbial communities present in different human body sites (Cho and Blaser, 2012). Predominant bacterial phyla present in both bovine (Figure 3) and human bodies (Cho and Blaser, 2012) include *Firmicutes*, *Proteobacteria*, *Actinobacteria*, and *Bacteroidetes*. Differences in bovine and human microbiota at specific anatomical side are expected at the bacterial genus level, as this is also the case with the mouse, in which 85% of the gut bacterial genera are not present in human gut (Ley et al., 2005). However, despite these highly significant differences, the mouse is still being used as an animal model to study human gut microbiome (Nguyen et al., 2015), whereas the bovine animal model holds several advantages over other animal models to investigate the role of the maternal microbiota (respiratory, gastrointestinal, and reproductive microbiota) in the DOHaD (Table 1). Considering the important implications of maternal respiratory, gastrointestinal, and reproductive microbiomes in child health and disease, we will discuss the microbiota of these three anatomic sites in bovine in the following sections. Of note, providing relatively detailed overview of the microbial compositions, and the sampling methods used to characterize these microbiotas in the following sections is for two reasons: (1) consideration of the readers who have little understanding of the bovine microbiome, and (2) for facilitating the better understanding of the proposed approaches to manipulate the maternal microbiota present in these sites.

#### Bovine Respiratory Microbiota

The bovine respiratory system consists of the upper (including nostrils, nasal cavity, and pharynx) and lower (containing the larynx, trachea, bronchi, and lungs) respiratory tracts. Multiple layers of defense mechanisms including physical, biochemical, and cellular barriers have evolved in bovine respiratory tract to counteract pathogen attachment rapidly and efficiently (Kato and Schleimer, 2007; Ackermann et al., 2010). Commensal microbiota residing within the bovine respiratory tract has recently been recognized as an additional safeguard and regulator of pulmonary defense against respiratory infections (Zeineldin et al., 2019; Timsit et al., 2020). Given the importance of the bovine respiratory disease (BRD) in feedlot cattle, investigation of the bovine respiratory microbiota using next-generation sequencing techniques has been extensively carried out in recently weaned beef calves (approx. 6 months after birth) during pre- and post-feedlot placement. Therefore, in the following sections we will describe the respiratory microbiota in beef cattle.

##### Upper Respiratory Tract Microbiota

Given its accessibility and by being the primary niche for opportunistic respiratory pathogens to colonize and proliferate, as well as being colonized by microbiota that is similar to lung microbiota (McMullen et al., 2020a), the nasopharynx of cattle has been the primary target for characterizing the microbial community in the respiratory tract. The nasopharyngeal (NP) microbiota of feedlot cattle contains a rich and diverse bacterial community, harboring approximately 29 phyla and 300 genera (Timsit et al., 2016). *Proteobacteria*, *Firmicutes*, *Actinobacteria*, *Bacteriodetes*, and *Tenericutes* are the predominant phyla and constitute over 90% of the total NP microbiota (Holman et al., 2015a,b, 2017; Zeineldin et al., 2017a). The most common genera include *Corynebacteria*, *Moraxella*, *Mycoplasma*, *Pasteurella*, *Mannheimia*, *Psychrobactor*, and *Staphylococcus* (Holman et al., 2015a, 2017; Zeineldin et al., 2017a; Timsit et al., 2018). The proportions of different bacterial communities in the NP vary between individual animals over time (Timsit et al., 2016).

##### Lower Airway Microbiota

Compared to the NP microbiota, the microbial community residing within the lower respiratory tract of finishing beef cattle has been less well characterized, partially due to the invasiveness and difficulty of sample collection. Transtracheal aspiration (TTA) and bronchoalveolar lavage fluid samples have mainly been used to characterize the lower airway microbiota of feedlot cattle. According to 16S rRNA gene sequencing of TTA samples obtained from these studies, a diverse and self-sustainable microbial community is present in the trachea of cattle, with colonization by species within the *Firmicutes*, *Proteobacteria*, and *Actinobacteria* phyla. The most relatively abundant genera include *Mycoplasma* (>50%), *Moraxella*, *Pasteurella*, *Lactococcus*, *Histophilus*, and *Bacteroides* (Nicola et al., 2017; Stroebel et al., 2018; Timsit et al., 2018; McMullen et al., 2020b). Similar to the tracheal microbiota, bronchoalveolar microbial communities from healthy feedlot cattle contained mostly *Proteobacteria*, *Bacteroidetes*, *Actinobacteria*, and *Tenericutes* (Zeineldin et al., 2017a; McMullen et al., 2020a). Overall, most of the predominant phyla and genera identified from the tracheal (Nicola et al., 2017; Timsit et al., 2018; McMullen et al., 2020a) and bronchoalveolar (Zeineldin et al., 2017b; McMullen et al., 2020a) samples are present in NP samples from the same cattle. A recent study characterized the microbial communities present in 17 habitats across upper and lower respiratory tract in healthy feedlot cattle and revealed that the microbial community structure and composition of NP microbiota are most similar to that of the lung microbiota (McMullen et al., 2020a). However, due to the difference in physiological gradients including the pH, relative humidity, temperature, and partial pressure of oxygen and carbon dioxide along the different parts of the respiratory tract (Man et al., 2017), the presence and relative abundance of certain bacterial taxa (e.g., Phylum *Tenericutes* and genera *Mycoplasma*) are highly abundant in the lower airway microbial community compared to NP microbiota (McMullen et al., 2020a). In addition, the lower respiratory microbiota exhibits lower bacterial richness (number of taxonomic groups) and evenness (distribution of abundances of the groups) compared to upper respiratory microbiota (Nicola et al., 2017; Timsit et al., 2018).

#### Perspectives on Using Cattle to Study the Maternal Respiratory Microbiota and Its Role in DOHaD

Although pregnancy-associated changes in the maternal gut microbiome have been relatively well documented, the impact of pregnancy on the microbial communities residing within the respiratory tract is much less well documented. Respiratory infections during pregnancy are common illnesses that contribute to pregnancy complications and increased antibiotic consumption during pregnancy (Laibl and Sheffield, 2006; Namazy et al., 2016). Multiple lines of evidence indicate that respiratory infection during pregnancy may have adverse effects on fetal (Siston et al., 2010; Englund and Chu, 2018) and neonatal (Doyle et al., 2013) development (Parker et al., 2016). In addition, the contribution of the mucosal microbiota to respiratory health as a gatekeeper (O’Dwyer et al., 2016; Man et al., 2017; Wypych et al., 2019) and its potential involvement in the gut-lung axis (Budden et al., 2017; Enaud et al., 2020) have been increasingly recognized. Therefore, it is important to characterize the dynamics of respiratory microbiota during pregnancy and identify potential factors regulating such dynamics.

To understand the longitudinal changes in maternal respiratory microbiota in response to pregnancy, the use of more intensive NP swab sampling or tracheal wash sampling is needed and is feasible in cattle. In addition, alteration of the respiratory microbiota using either antibiotics or probiotics will enable researchers to induce targeted changes in the maternal respiratory microbiota during pregnancy, uncovering the role of pulmonary microbiota in fetal programming and offspring respiratory health. Amat and colleagues successfully induced a longitudinal alteration in NP microbiota of newly weaned beef calves (7–8 months postpartum) *via* intranasal inoculation of bacterial therapeutics (Amat, 2019). In this study, inoculation of a single dose of intranasal bacterial therapeutics consisted of six *Lactobacillus* strains that were able to alter microbial community structure and composition, and the species–species interaction network of NP microbiota for up to the end of 42 days monitoring period. The authors also compared the efficacy of bacterial therapeutics with a commonly used metaphylactic antibiotic, tulathromycin. Following antibiotic administration, the NP microbiota exhibited significant changes in both alpha and beta diversity, as well as interaction networks (Amat, 2019). Antibiotic-induced alterations in bovine NP microbiota were distinctively different from the changes induced by intranasal bacterial therapeutics. Thus, the results of this study suggested that probiotic and antibiotic approaches can be applied to deliberately induce dramatic changes in maternal respiratory microbiota, which could allow a better understanding of the role of the maternal respiratory microbiota in fetal programming and offspring respiratory health.

Pathogen challenge studies can be used to investigate whether the interventions in maternal microbiota during pregnancy can have long-term impacts on respiratory disease resilience in offspring. Along these lines, several studies have demonstrated the ability to induce BRD symptoms in weaned and yearling cattle. For example, Kayser et al. (2019) were able to challenge 11-month-old beef crossbred Angus steers by bronchoselective endoscopic inoculation with 10 ml of *M. haemolytica* serotype A1 [1.2–1.4 × 10^9^ colony-forming units (CFU) per ml]. Following the *Mannheimia haemolytica* challenge, the steers experienced acute-immune responses and behavioral changes that are synonymous with naturally occurring BRD. Likewise, both BRD bacterial (*M. haemolytica*, *Pasteurella Multocida*, and *Histophilus somni*) and viral pathogen challenges have been successfully demonstrated in 4- to 6-month-old steers (Angus-Hereford cross; Gershwin et al., 2015). These studies together suggest that it is feasible to challenge pregnant female cattle with respiratory infectious agents during pregnancy, and thereby study the impact of pathogen challenge-induced changes in maternal respiratory microbiota on fetal programming and offspring respiratory health.

Conducting a challenge study in offspring (calves) to identify the impact of maternal microbiota/dietary alterations on offspring respiratory health is also feasible. Pathogen challenge studies have been most frequently performed in neonatal and suckling calves (Riffault et al., 2010; Lhermie et al., 2016; Amat et al., 2020). The pathogens used to challenge the calves include both a mono-inoculation of *M. haemolytica*, or other respiratory bacterial pathogens in conjunction with BRD viral pathogens. Pathogen inoculation is accomplished by either intranasal (Amat et al., 2020) or intratracheal (Riffault et al., 2010; Lhermie et al., 2016) routes. Conducting challenge studies in the offspring will provide more direct evidence to test the hypothesis pertaining to the role of maternal microbiome in defining offspring health or disease resilience.

#### Bovine Gut Microbiota

The bovine gastrointestinal tract microbiome is important in maintaining animal health and production. It encompasses the microbial communities residing with the rumen (known as the ruminal microbiota) and also the lower-gut, which consists of both small intestine and the hindgut regions (O’Hara et al., 2020). The diversity of the microorganisms that colonize bovine GIT includes bacteria, archaea, protozoa, fungi, and viruses (Matthews et al., 2019; O’Hara et al., 2020). The recent application of high-throughput sequencing allows for better understanding of the microbial composition and function of these microbiota, and their role in maintaining cattle production and health, which is still a challenge to accomplish using the conventional culture-dependent approaches (Huws et al., 2018).

##### Ruminal Microbiota

The bovine rumen functions as a unique and highly specialized ecosystem, teeming with a variety of microorganisms that have cellulolytic, semi-cellulolytic, amylolytic, proteolytic, and lipolytic activities (Matthews et al., 2019; O’Hara et al., 2020). These ruminal microorganisms are critical for providing nutrients to the host by degrading host-indigestible, plant-based feedstuffs (Matthews et al., 2019; O’Hara et al., 2020). Bacteria are the most densely populated microorganisms in the rumen, with over 200 species and a cell density ranging from 10^10^ to 10^11^ cells per ml of ruminal content (Matthews et al., 2019). The ruminal bacterial community is dominated mainly by the seven phyla: *Proteobacteria*, *Bacteroidetes*, *Firmicutes*, *Spirochaetes*, *Fibrobacteres*, *Verrucomicrobia*, and *Tenericutes* (Holman and Gzyl, 2019; Bailoni et al., 2021). The core bacterial genera present in the rumen are *Fibrobacter*, *Prevotella*, *Ruminococcus*, and *Succiniclasticum* (Jami et al., 2013; Holman and Gzyl, 2019). The second most densely populated microorganisms in the rumen are methanogenic archaea, accounting for 4% of the microbial community (Matthews et al., 2019). The ruminal archaeal community is mainly dominated by the genera *Methanobrevibacter* (63.2% of methanogen population), *Methanomicrobium*, *Methanosphaera*, *Thermoplasma*, and *Methanobacterium* (1.2%; Matthews et al., 2019). *Methanobrevibacter* and *Methanosphaera* are reported to be core methanogenic genera in the bovine rumen (Holman and Gzyl, 2019). These methanogenic archaeal populations utilize fermentation end-products and produce methane. In addition to bacterial and archaeal microbial communities, the bovine rumen is also inhabited by ciliated protozoa, fungi, and bacteriophage, all of which are critical in ruminal fermentation and nutrient metabolism in the rumen (Matthews et al., 2019). The composition and functional characteristics of protozoa, fungi, and bacteriophages have been relatively less characterized compared to ruminal bacteria and archaea. A partial reason for this is due to the relative ease of utilizing high throughput sequencing to quantify archaeal and bacterial 16S rRNA genes (Holman et al., 2017). The community structure and composition of the microbial community in the rumen are dynamic, and our current understanding is that they are influenced mainly by age and diet (Jami et al., 2013; Matthews et al., 2019). These latter observations are interesting as developmental outcomes also are influenced strongly by maternal age and diet (Reynolds and Caton, 2012; Reynolds et al., 2019, 2022).

##### Lower-Gut Microbiota

The microbial community residing within the lower gut regions is less densely populated, and microbial community structure is less diverse compared to that of the ruminal microbiota. This is partially due to the fact that the microbial fermentation taken place in the hindgut is responsible for less than 30% of total tract cellulose and hemicellulose degradation (O’Hara et al., 2020). The bacterial microbiota residing within the lower-gut is phylogenetically similar to the rumen microbiota and is predominately colonized by *Firmicutes* and *Bacteroidetes*, with *Prevotella*, *Ruminococcus*, *Lachnospiraceae* UGG-008, and *Eubacterium* being core genera in the regions of jejunum, cecum, and colon (Holman and Gzyl, 2019; O’Hara et al., 2020). In the lower gut, the microorganisms are involved in nutrient metabolism but in addition play a critical role in immune system development (O’Hara et al., 2020).

#### Perspectives on Using Cattle to Study the Maternal Gut Microbiota and Its Potential Involvement in DOHaD

Although it has been relatively well documented that the maternal gut microbiota in pregnant women undergoes dramatic changes in microbial composition and function over the course of pregnancy in response to the increased metabolic demands of the developing fetus (Collado et al., 2008; Koren et al., 2012; Smid et al., 2018; Codagnone et al., 2019a), some fundamental questions pertaining to the mechanisms underlying the regulation of the maternal gut microbiota response to pregnancy remain. The bovine animal model has the potential to be a superior biomedical model for studying the changes in the maternal gut microbiota throughout the course of pregnancy and identifying the underlying mechanisms involved in pregnancy-associated maternal gut microbiome changes. Despite the difference in microbial population in the bovine GIT, the anatomy and physiology of the gut between bovine and human, the similarity in terms of gestational age (40 weeks), singleton pregnancy, reproductive aging (Malhi et al., 2005), preimplantation development of embryos, embryogenesis (Simmet et al., 2018), and a shared genetic architecture of complex traits, suggest that the data obtained from the bovine animal model regarding maternal microbiota and factors involving in the regulation of maternal gut microbiota will be more valuable in directing human maternal medicine compared to the data obtained from rodent based studies. For example, reproductive technologies readily implemented in bovine models allow for standardization of targeted breeding dates, ability to utilize a single sire for all offspring, ability to target sex of offspring *via* sex-sorted semen, and the ability to generate multiple offspring from a single sire/dam combination *via* multiple ovulation embryo transfers (Dahlen et al., 2014). Estrous synchronization and artificial insemination, a common means of breeding cattle, enables conception, and therefore parturition, within a relatively tight window of time. Reduced variation in the time of conception and calving and similarities in sex and parentage of offspring within a study population will minimize the variations in both maternal and offspring microbiota. Additionally, longitudinal monitoring of the maternal gut microbiota before and during pregnancy, and direct comparison of the maternal gut microbiota changes in pregnant vs. non-pregnant cohort females is also feasible in cattle, which is critical to uncover how the maternal gut microbiota changes in response to pregnancy. Additionally, ruminal fluid samples can be collected from pregnant cattle over the course of pregnancy using a relatively non-invasive ruminal tubing method (Paz et al., 2016; Amat et al., 2021b). Ruminal sampling *via* tube was performed on heifers during the 2nd and 6th months of pregnancy and had minimal adverse effect on the heifers (Amat et al., 2021b). Longitudinal sampling of ruminal fluid and feces collected *via* rectal grab sampling (Fredin et al., 2014) will provide more comprehensive characterization of the gut microbiota changes during pregnancy.

In addition, it is possible to perform targeted manipulation of the bovine gut microbiota during pregnancy *via* altering the diet composition (high forage or high grain diet) while maintaining the nutrient intake balance, or altering maternal feed intake (Diniz et al., 2021a; Menezes et al., 2021), or feeding direct-fed microbes (Jeyanathan et al., 2016, 2019; Philippeau et al., 2017) or probiotics (Uyeno et al., 2015; Cameron and McAllister, 2019), or oral administration of antibiotics (Chen et al., 2020). The recent advanced feeding systems including an electronic feeding system (Insentec, BV, Marknesse, the Netherlands), Super smartFeed (C-Lock, Rapid City, South Dakota, United States), and GrowSafe Systems allow for individual control of feeding and monitoring of daily feed intake and collection of more comprehensive data regarding the cattle behavior and daily activities. In addition, pregnant beef cattle are often housed in less confined but more natural environments (Rørvang et al., 2018), which can be challenging to do with rodent laboratory animals.

#### Bovine Reproductive Tract Microbiota

Emerging lines of evidence derived from human and invertebrate animal models suggest that microbiomes residing within the reproductive tract may influence reproductive efficiency, and that microbiome-targeted approaches may provide a novel opportunity to reduce the incidence of reproductive failures (Koedooder et al., 2019; Rowe et al., 2020; Tomaiuolo et al., 2020). Next-generation sequencing techniques have recently enabled the characterization of the microbial communities residing within different parts of bovine female reproductive tract, and microbial habitants have helped identify the role of the microbiome in reproductive tract health and fertility. Within the female bovine reproductive tract, vaginal and uterine microbiota have been relatively well characterized. Microbial communities present in the cervix and placenta have also been characterized but to a lesser extent compared to vaginal and uterine microbiotas.

##### Vaginal Microbiota

The bovine vagina harbors a diverse and distinctive microbial community, differing from the microbial community residing within the upper reproductive tract. The vaginal microbiota is predominantly colonized by facultative anaerobic bacteria affiliated with the phylum *Firmicutes* (accounting for 40–50% of the total vaginal bacterial population). The second most predominant phylum present in the vagina; however, is the anaerobic *Bacteroidetes* (15–25%). *Preoteobacteria* (facultative anaerobic), *Actinobacteria*, *Euryarchaeota*, and *Tenericutes* are also dominant phyla in the vaginal tract (Laguardia-Nascimento et al., 2015; Yeoman et al., 2018; Deng et al., 2019; Galvão et al., 2019). There are more than 300 genera present in the vaginal microbiota of beef cows (Swartz et al., 2014), with *Ureaplasma*, *Ruminocuccus*, *Streptococcus*, *Fusobacterium*, and *Porphyromonas*, being most predominant (Laguardia-Nascimento et al., 2015; Galvão et al., 2019). It has also been reported that the vaginal microbiota of virgin heifers is diverse and dynamic, and undergoes significant changes during the estrous cycle (Quereda et al., 2020). The vaginal microbiota of virgin heifers is slightly different in terms of microbial community structure and composition from that of mature cows (Laguardia-Nascimento et al., 2015), perhaps due to contamination during the periparturient period. Interestingly, some of the taxa (e.g., *Ruminococcus*, *Prevotella*, *Bacteroides*, and *Clostridium*) present in vaginal tract are also commonly found in the rumen of cattle and are key members of the ruminal microbiota (Laguardia-Nascimento et al., 2015). Of note, the vaginal microbiota in healthy women is dominated by various *Lactobacillus* spp (Martin, 2012; Barrientos-Durán et al., 2020). In the bovine vagina, *Lactobacillus* species are not as dominant as in the human vagina (Swartz et al., 2014). The relationship among ruminal and vaginal microbiome warrants further investigation as no direct anatomical link between these two locations exists. Though oral inoculation from licking of other cattle (a relatively common occurrence related to behavior during estrus) could be a mechanism of transfer, movement of microbiota through circulation is a potential alternative mode of transfer. The presence of bacterial microbiota in peripheral blood cells (PBMC) has recently been reported in small ruminants (Peña-Cearra et al., 2021). Thus, it is plausible that circulating microbiota from the dam could certainly inoculate a developing fetus.

##### Cervical Microbiota

Whereas pathogenic bacterial colonization in the bovine cervix has been relatively well documented, recent next-generation sequencing-based studies have revealed that the cervix harbors a distinct microbial community that differs from that of the vagina and uterus. Overall, there is a relatively rich microbial community present in the cervix of healthy cows regardless of physiological stage, harboring at least 1,000 taxa (Wang et al., 2018). The top five most abundant phyla of the cervical microbiota are *Bacteroidetes*, *Proteobacteria*, *Firmicutes*, *Tenericutes*, and *Actinobacteria* phyla. The most predominant genera include *Porphyromanas*, *Fusobacterium*, *Fontimonas*, *Ruminococcaceeae*_UCG005, *Bacteroides*, and *Pseudonomas*.

##### Uterine Microbiota

Due to the significant economic and animal welfare impact of uterine infections such as metritis and endometritis, which are important reproductive disorders in both dairy and beef cattle (Dohmen et al., 2000), characterization of the uterine microbial community has long been a focus of research and was traditionally focused exclusively on the pathogenic bacteria using traditional culture-based approaches. Although the vagina is home to billions of bacteria, the *in-utero* environment has long been believed to be sterile as the cervix is equipped with layers of barriers that prevent ascending of bacteria from the lower reproductive tract into the uterus. The presence of bacteria *in utero* is thus considered as contamination due to a compromised/damaged cervical barrier. However, recent advancements in culture-independent, high-throughput sequencing technologies enabled the identification of commensal microbiota presence in the bovine uterus during both pregnancy and after parturition (Galvão et al., 2019).

Santos et al. (2011) first applied a culture-independent method, which was a clone library sequencing-based metagenomic analysis to evaluate and compare the uterine bacterial composition in Holstein dairy cows with uterine infections (metritis) and those that were healthy. They reported that the uterine microbial composition was much more diverse, and complex compared to what had been observed by using culture-based methods. Overall, *Gammaproteobacteria*, *Firmicutes*, *Fusobacteria*, *Bacteroidetes*, and *Tenericutes* were the most abundant phyla observed in the intrauterine fluid samples across all dairy cows. The same authors then used a combined PCR-DGGE and 454 pyrosequencing to gain deeper insights into the uterine microbiota of healthy or metritic and endometritic Holstein dairy cows at three intervals following calving (Santos and Bicalho, 2012). In this study, *Fusobacteria* (34.3%), *Bacteroidetes* (29.1%), *Proteobacteria* (12.5%), *Firmicutes* (12%), *Tenericutes* (7.7%), and *Actinobacteria* (1.3%) were identified as the main bacterial phyla across all uterine fluid samples.

After these studies, the application of 16S rRNA gene sequencing on the Illumina MiSeq platform enabled a more comprehensive survey of the community structure and diversity of the uterine microbiota. Jeon et al. (2015) investigated the progression of the uterine microbiota from calving until the establishment of metritis in Holstein dairy cows. They identified 28 phyla and 824 genera across all uterine swab samples. More than 98% of the uterine microbiota was constituted by six phyla: *Bacteroidetes* (28.9%), *Proteobacteria* (22.5%), *Fusobacteria* (20.2%), *Firmicutes* (15.8%), and *Tenericutes* (10.7%). At the genus level, *Fusobacterium* (15.7%) was the most abundant genus, followed by *Bacteroides* (13.9%), *Coxiella* (12.7%), *Porphyromonas* (9.9%), and *Ureaplasma* (5.2%). Most of these predominant phyla and genera were identified in the uterus of healthy dairy cows within 8 days postpartum (Jeon et al., 2015). It is commonly believed uterine colonization by a microbial population happens following parturition as the cervical defense mechanisms against microbial translocation from vagina to uterus is compromised or the reproductive tract is damaged due to the birthing process. However, recent studies revealed that the microbial community in the uterus is present during pregnancy, and pregnancies are established and maintained in the presence of a uterine microbiota (Karstrup et al., 2017; Moore et al., 2017). The uterine microbiota of virgin heifers and its community structure and composition changes during the estrous cycle have not been characterized yet.

#### Perspectives on Using Cattle to Study Maternal Reproductive Microbiota and Its Role in DOHaD

Given that the reproductive microbiota is important in reproductive health and pregnancy, it is important to understand the dynamics of the microbial community in the reproductive tract (vagina, cervix, uterus, and oviduct) in response to pregnancy, and factors regulating such changes. A longitudinal survey of bovine reproductive microbiota at the time of breeding and during pregnancy can provide important information that can be translated into human maternal practices. Vaginal swab (Amat et al., 2021b), cervical swab (Wang et al., 2018), uterine swab (Jeon et al., 2021), and endometrial brush (Pascottini et al., 2020) sampling from pregnant cattle allow monitoring of the changes in microbiota residing in each of the anatomical sites along the reproductive tract. Applying targeted alterations of vaginal and uterine microbiota during early or late gestation with probiotics or antibiotics will allow for studying the impact of maternal microbiota on pregnancy and health of the dams, and on fetal programming and offspring development. An additional paradigm to consider is the feasibility of surveying multigenerational impacts of maternal reproductive microbiota alterations during pregnancy on offspring reproductive microbiome development and reproductive efficiency. Though studies to investigate the multigenerational impact of maternal nutrition during early gestation on offspring development and health have been conducted in beef cattle (McLean et al., 2017; Amat et al., 2021b; Diniz et al., 2021b; Menezes et al., 2021), whether multigenerational impacts exist on microbiota remains to be elucidated. With the common application of sexed semen in cattle production (Hohenboken, 1999), the sex of the fetus can be controlled, which allows the selective production of female or male offspring when studying the reproductive microbiota and its multigeneration impact. The F1 generation female offspring (calves) can be bred at around 14–15 months of age and will give birth to the F2 generation female offspring at around 2-years of age. Thus, within a several year time span, the impact of the maternal reproductive microbiota on several generations of offspring can be studied using the human-relevant bovine model.

## Using the Bovine Model to Study *in utero* Microbial Colonization

Despite increasing evidence derived from human and bovine studies suggesting the existence of prenatal microbial colonization (D'Argenio, 2018; Stinson et al., 2019; Guzman et al., 2020; He et al., 2020; Rackaityte et al., 2020; Husso et al., 2021; Mishra et al., 2021; Amat et al., 2022), the “*in utero* microbial colonization hypothesis” still remains as a controversial subject, with many investigators supporting the “sterile-womb hypothesis” (Perez-Muñoz et al., 2017; de Goffau et al., 2019, 2021; Walter and Hornef, 2021). Those not in support of the “*in utero* microbial colonization hypothesis” argue that the limitations associated with the molecular approaches are insufficient to detect “low-biomass” microbial populations in fetal intestine, lack of contamination controls, and failure to provide viable microbes, thereby making the findings of the studies that reported the presence of microbes in fetal intestine weak (Perez-Muñoz et al., 2017). It is also argued that anatomical, immunological, and physiological characteristics of the placenta and fetus prevent *in utero* microbial colonization. Also, skepticism about prenatal microbial colonization stems from the generation of axenic animals and humans (Perez-Muñoz et al., 2017). Thus, it is pivotal to provide more convincing and robust evidence that either supports or refutes the “*in utero* microbial colonization hypothesis,” as this has critical implications for current clinical practices that are focused on the prevention of microbiome perturbations primarily at and (or) after birth and their role in DOHaD.

Fewer ethical restrictions were placed on farm animal models than on studies with human subjects, with the feasibility of sampling the fetus at different gestational ages, and the greater tissue and fetal fluid availability from the bovine fetuses make the bovine animal model more appealing to study this topic. Investigating microbial presence in gravid uteri (i.e., containing maternal uterine tissues, fetus, placenta, and associated fluids) that are obtained at different developmental stages not only provides evidence for the *in utero* microbial colonization, but also allows identification of the timeline for pioneer intestinal microbial colonization. For example, we recently obtained gravid bovine uteri at 63-days (Amat et al., unpublished data) and 84-days (Amat et al., 2022) of gestation to investigate the presence of bacterial microbiota *in utero*-placental tissues in early gestation. Fetuses that were obtained at mid to late gestation (5–7 months; Guzman et al., 2020) and near-full term (Husso et al., 2021) were also investigated for the prenatal microbial population.

Given that there is low microbial biomass in gravid uteri samples, which often compromises the detection *via* the sequencing-based methods, it is necessary to use a larger volume of fetal fluid (amniotic or allantoic fluid) and larger pieces of fetal intestinal samples for genomic DNA extraction. Bovine pregnancies at day 50 of gestation typically provide about 10–15 ml of amniotic fluid (Crouse et al., 2019). Up to 20–30 ml, amniotic or allantoic fluid samples can be obtained from bovine pregnancies at day 60 of gestation (approx. BW = 18–19 g; Ward et al., unpublished data; Figure 2), and this volume is approximately doubled when the fetus is 84 days of gestation (Amat et al., 2022). Besides, the relatively larger fetal intestine provides the option of using larger pieces of intestinal tissue for genomic DNA extraction or for culturing or microscopic imaging. Fetal fluid samples can be obtained from the intact fetus using a sterile preparation, syringes, and needle without exposing the contents of the gravid uterus to the environment (Amat et al., 2022), which minimizes the potential contamination of the fetal fluids from maternal, fetal, or environmental surface exposure during delivery or at the time of specimen transport. To account for any potential contamination of the fetal intestine, swab samples from the utero-placenta, surface of the fetus, and the surgical trays, tables, and instruments and room air can be collected. Thus, the approaches discussed above are expected to overcome some of the concerns regarding the lower microbial biomass and potential contamination of fetal samples and allow for true evaluation of microbiome presence in specific uterine, placental, and fetal compartments.

In addition to profiling the fetal microbiota using sequencing- or culture-based approaches, providing indirect evidence for the presence of *in utero* microbial colonization can be achieved in cattle *via* infusion of a broad-spectrum antibiotic directly into the uterus on different gestational stages. Intrauterine antibiotic infusion can be done using ultrasound-guided transvaginal amniocentesis (Garcia and Salaheddine, 1997) during the first and 2nd trimester, whereas antibiotic infusion into the uterus can be accomplished *via* laparoscopy during the 3rd trimester. In addition, it is feasible to deliver certain heat-killed pathogens or live commensal bacteria directly into the uterus using these methods. Such approaches will help to uncover the role of the feto-maternal microbial crosstalk in developmental programming, development of the microbiome in offspring, and long-term consequences for offspring health. These approaches are challenging or difficult to do with laboratory animals.

### Challenges Associated With the Bovine Animal Model to Study the Role of the Maternal Microbiome in Fetal Programming and Offspring Development

When using the bovine animal model to study the role of the maternal microbiome in fetal programming and offspring development, the following challenges may arise. First, the larger expenses required for purchase and maintain the animals. The cost varies depending on the age, body condition, and specific dietary targets of the female cattle, but is expected to be significantly greater than the cost associated with sacrificing a pregnant mouse, sheep, or pig. The greater cost of bovine animals compared to other animal species; however, may well be justified as this biomedical model is more likely to provide valuable information for the development of maternal microbiome targeted strategies to improve human health and well-being. The second challenge associated with the use of bovine animals to study perinatal microbial colonization lies with the difficulty of creating and maintaining an aseptic environment to guarantee the collection of uteroplacental and fetal tissue samples under sterile conditions. Uteroplacental and fetal tissue samples are expected to have low microbial biomass, making it difficult to detect and isolate a very sparse bacterial signal from both background noise and mammalian signal. Therefore, it is critical to use robust aseptic techniques during the slaughter of the dams and fetal sampling to minimize potential contamination. In addition, collecting rigorous contamination control samples is also required to account for contaminant microbes. Third, the key difference in digestive systems of bovine and humans may cause difficulties in interpreting the results of experiments conducted to investigate the maternal gut microbiota and its involvement in fetal programming and offspring microbiome development. The gut microbiota of the bovine which is a ruminant animal having a complex stomach with four compartments is more densely populated with microorganisms that contain cellulosic enzymes to digest the cellulosic compounds in plant matter (Matthews et al., 2019) compared to the gut microbiota of humans whose stomach has only one chamber that evolved to digest both plant- and animal-derived foods (Smith and Morton, 2010). Furthermore, rodent animals can be housed in small cages and the housing facilities can often be in the same building with the research laboratories. Whereas, large animals including bovine require bigger space and natural environments, and thus, they need to be housed in facilities located relatively remotely from where the research labs are located. Finally, experts who have a solid background in human biomedical research and human microbiome may have training limited to rodent animal models and therefore have limited understanding of the physiology, microbial ecology, and animal husbandry of large animals including bovine (Polejaeva et al., 2016). As a result, the use of bovine animals in biomedical research is less common than the rodent animal models.

## Conclusion

Despite these challenges discussed above, it is more likely that the bovine model will become an increasingly important animal model for research to identify the timeline of the pioneer microbial colonization and explore the role of the maternal microbiome in fetal and postnatal organ development and, ultimately, DOHaD. The unique advantages that the bovine model possesses (outlined throughout this review) still make this model superior to other farm and rodent animal models. These advantages include: having singleton pregnancy and similar gestation period to humans; insemination with sexed semen; feasibility of targeted alterations of the maternal respiratory, gastrointestinal and reproductive microbiota using prebiotics or antibiotics; accessibility of large amounts of fetal tissue and fetal fluid samples even at early gestation; the possibility of sampling the fetus at different stages of gestation and conducting microbially-targeted interventions within the utero-placenta *via* antibiotic infusion. As mentioned, these approaches are challenging or impossible to do with rodent animal models. Thus, investigation of the involvement of the maternal microbiota in DOHaD using bovine animal model will provide important information that will have significant implications for the practice of maternal, fetal, and pediatric medicine in both humans and animals.

## Author Contributions

SA: conceptualizing and outlining the review, conducting the literature search, and drafting the initial manuscript. KS, CD, AW, LR, JC, and SA: manuscript writing, revision, editing, and finalizing. All authors have read and agreed to the published version of the manuscript.

## Funding

SA is supported by start-up funding from the North Dakota Ag Experiment Station, and funding from the USDA National Institute of Food and Agriculture (Hatch project number ND02440).

## Conflict of Interest

The authors declare that the research was conducted in the absence of any commercial or financial relationships that could be construed as a potential conflict of interest.

## Publisher’s Note

All claims expressed in this article are solely those of the authors and do not necessarily represent those of their affiliated organizations, or those of the publisher, the editors and the reviewers. Any product that may be evaluated in this article, or claim that may be made by its manufacturer, is not guaranteed or endorsed by the publisher.
